# Nursing Students’ Education During COVID-19 Pandemic

**DOI:** 10.31661/gmj.v9i0.2033

**Published:** 2020-12-29

**Authors:** Reza Heidari-Soureshjani, Golrokh Abdolahi, Fariba Tabari

**Affiliations:** ^1^Students’ Scientific Research Center, Faculty of Nursing and Midwifery, Tehran University of Medical Sciences, Universal Scientific Education and Research Network (USERN), Tehran, Iran; ^2^Department of Medical-Surgical Nursing and Basic Sciences, School of Nursing and Midwifery, Tehran University of Medical Sciences, Tehran, Iran; ^3^School of Nursing and Midwifery, Tehran University of Medical Sciences, Tehran, Iran

**Keywords:** COVID-19 Pandemic, Nursing Students, Education

## Dear Editor,

COVID-19 is the name of an infectious disease called Coronavurus 2019, one of the causes of respiratory failure; The disease was first identified in Wuhan, China, in December 2019, and then spread around the world and also creating the 2019-2020 coronavirus pandemic [1,2]. The disease is currently reported in 215 countries, with an estimated more than 8 million cases [3]. Due to the high prevalence of COVID-19, rapid transmission, mortality in severe cases, and the absence of definitive drugs, the disease is a significant threat to human life and health that can affect various aspects of physical and mental health [4–6].

Creating emotional problems and mental disorders such as stress, anxiety, and fear is one of the inevitable consequences of this disease that can cause stress, depression, and even suicide. Given that students, as a critical element of the educational system, have a unique role in achieving the educational system, it is essential to pay attention to their mental, emotional and physical health [1,7,8]. Most universities around the world are closed to prevent the spread of new virus infections. Furthermore, this quarantine and closure of universities can hurt various students’ educational process, especially nursing students who have practical hospital courses. Due to universities’ closure in most of the areas affected by the COVID-19, face-to-face training of students has been suspended temporarily. Many measures have been taken for effective virtual education. However, these measures’ effectiveness is not the same for all students because students study several theoretical and practical courses in semesters. However, some point out that virtual learning for students with theoretical courses can be useful because there is no need to wake up early. There is no fear of getting scolded, and one can study in a comfortable situation. However, it should be noted that this manner is not a substitute for classroom learning because, in the classroom, the communication and interaction between teachers and students are more robust [9–13].

 Despite the significant advancement of virtual learning globally, due to courses’ practicality in the higher semesters of nursing students, virtual education can have adverse effects on how they learn. On the other hand, nursing internship students, due to their close interaction with the hospital are affected by various conditions such as: Fear of being a Coronavirus carrier and its transmission to the family, high job fatigue, reduced sleep quality, malnutrition, fear of death, lack of proper communication with the family, frustration, Violent Behavior, etc.

Also, in some studies, students’ anxiety and the COVID-19 pandemic were higher than the national norm [11,14]. So, concerning the nursing community is essential to at the time that they are directly fighting this disease. Due to the job prospects of many nursing students depend on this. The ignoring of nurses needs could create a pessimistic vision for the nursing students, followed by a lack of interest in education, academic decline, dropping out of school, changing the field, etc.

It is also essential to consider new approaches to teach clinical skills. Due to the clinic’s distance, hospital, and eye to eye contact with patients, nursing internship, students have practical skill gaps. Therefore, encouraging students to use educational multimedia, clinical databases may be effective [15]. Finally, we suggest that nursing education managers need to take the necessary measures to ensure the safety of clinical education and consider and monitor the quality of virtual education for students.
